# Editorial: Neuroinflammation and the Visual System

**DOI:** 10.3389/fneur.2021.724447

**Published:** 2021-08-13

**Authors:** Gemma Caterina Maria Rossi, Claudia Angela Michela Gandini Wheeler-Kingshott, Ahmed Toosy

**Affiliations:** ^1^Fondazione Ospedale San Matteo (Istituto di Ricovero e Cura a Carattere Scientifico), Pavia, Italy; ^2^Department of Surgical Sciences, University Eye Clinic of Pavia, Pavia, Italy; ^3^Faculty of Brain Sciences, Queen Square Institute of Neurology, University College London, London, United Kingdom

**Keywords:** neuroinflammation, glaucoma, diabetic retinopathy, optic neuritis, multiple sclerosis, neuromyelitis optica spectrum disorders, microglia, OCT

The eye is an extension of the central nervous system (CNS) and degenerative diseases of the retina and optic nerve can lead to progressive loss of vision. The causes of degeneration are different and can overlap between genetic predisposition, environmental factors, metabolic alterations, and inflammatory processes.

New diagnostic methods and biomarkers are needed to examine and identify the role of neuroinflammation in the degenerative diseases affecting the visual system, not only to aid early diagnosis but also to monitor neuroprotective treatments.

The purpose of the present Research Topic was to publish new research describing potential new advances in the diagnosis, treatment, and pathological understanding of conditions that possess inflammatory components affecting the eye and the visual central nervous system.

## Neuroinflammation and the Eye

Diabetic retinopathy and glaucoma are two of the most prominent causes of vision loss (1). The pathogenesis of these conditions is complex and not yet fully known but there is consensus on the crucial role of neuroinflammation together with the mechanical/ischemic determine the onset and progression of these two pathologies. Previous studies have shown that microglial cells play an active role in maintaining the normal structure and functioning of the retina and the CNS, but also that a chronic proinflammatory environment is a common and important denominator of retinal degenerative diseases and neurological disorders affecting the vision (2).

The mini-review on neuroprotection and glaucoma by Rolle et al. reminds us that inflammatory responses within the retina are regulated by microglia and astroglia Rolle et al. These cells include Müller cells and astrocytes and provide metabolic support to neurons, neurological regulation of ion concentrations, and neuroprotective activities. Microglial cells originate from primitive erythromyeloid progenitors; after maturation, they participate in the inflammatory process activated by DAMPs (damage-associated molecular patterns) released by neural cells and also by astroglia: heat shock proteins (HSPs) are produced by retinal ganglion cells (RGC) when intraocular pressure (IOP) is high. In response to the neuroinflammatory process, microglial cytokines and chemokines (complement factors and interleukin 6) amplify the response and promote the morphological change of microglia into macrophages. There are two phenotypes of activated macrophages, M1 and M2, which produce IL-1β, IL-12, TNF-α and IL-10, TGF-β, and neurotrophic factor insulin-like growth factors, respectively. The review points out that animal model studies have improved our understanding of cellular mechanisms and pathways of neuroinflammation, paving the way for new therapeutic possibilities to modulate neuroinflammation such as antioxidants, ketogenic diet, hydrophilic saffron extract.

Another review by Tirendi et al. describes recent developments related to the new 3D trabecular meshwork (3d-TM) culture model that uses microengineering techniques to create systems that mimic cell-cell and cell-environment interactions found *in vivo*. The authors have recently studied, as markers of activation of the inflammatory response, the changes in pro-inflammatory cytokine transcriptions and NF-kB protein levels following oxidative stress stimulation, allowing to analyze the phases of cellular damage that underlie glaucoma and its adverse outcomes.

The relationship between diabetes and inflammation is studied by Filippelli et al. in patients with proliferative diabetic retinopathy. In this condition, oxidative stress is promoted by several mechanisms, including pathways involving polyols, vascular dysfunction, proinflammatory cytokine, and protein kinase C production, accumulation of advanced glycation products, activation of the renin-angiotensin-aldosterone system, increased growth factors, and leukostasis. Several papers have reported an increase in the intravitreal concentration of the main proinflammatory cytokines and chemokines and have highlighted a key role of these mediators in the onset and progression of diabetic retinopathy. The study by Filippelli et al. examines the vitreous composition in patients with diabetic retinopathy and reports an *in vivo* protective role *in vivo* of some components of the diet (curcumin, omotaurine, and vitamin D3) that could be used in addition to anti-neovascularization agents, to reduce cytokine levels, to regulate the inflammation network and reduce the rate of administration of intravitreally injected agents. An additional gene expression experiment demonstrated that the combination of curcumin, vitamin D3, and homotaurine down-regulate the expression of the cyclinD1 gene and the proinflammatory cytokine genes TNFα and IL6, supporting their anti-inflammatory action in combination.

Finally, Tong et al. present an interesting resting-state functional magnetic resonance imaging study in patients with iridocyclitis and demonstrate altered and disturbed synchronous neural activity within certain areas of the brain, suggesting some form of neuroplasticity in this condition.

## Neuroinflammation and Central Nervous Visual System

Acute optic neuritis is a frequent manifestation of inflammatory CNS conditions, multiple sclerosis (MS), and neuromyelitis optic spectrum disorder (NMOSD), with the latter predisposing to significant and irreversible vision loss. Key research areas aim at understanding the disability and recovery mechanisms with acute neuroinflammation and neurodegeneration of the optic nerves in these conditions. This Research Topic presents a large selection of articles that enhance our understanding of these areas, but which can also be grouped into several important research themes.

The first theme concerns the use of animal models. Redler and Levy present a comprehensive review article on the application of rodent models of optic neuritis and how they contributed to our understanding of the pathological mechanisms of inflammation, demyelination, axonal loss, and therapies such as antioxidants, neuroprotective agents, and remyelinating agents. Another article by Dietrich et al. reports that dimethyl fumarate, a disease-modifying treatment commonly used in MS, has no protective effects on retinal degeneration after optic nerve crushing in mice, but appears to exhibit antioxidant and anti-inflammatory effects after light-induced photoreceptor loss.

The second main theme concerns biomarkers, both imaging and immunopathogenic. For imaging, articles related to optical coherence tomography (OCT) dominate this Research Topic. Kleerekooper et al. present an excellent review of the recently available imaging modality, OCT angiography, capable of visualizing retinal and choroidal vasculature in high detail with the promise of providing new insights into the pathobiology of MS and NMOSD. Ziccardi et al. report an application of OCT to MS patients with a history of optic neuritis (ON) and find that post-ON neurodegeneration affects both the outer and inner retinal layers in patients with poor visual recovery whereas it occurs only in the inner retinal layers with good visual recovery. Murphy et al. report significant correlations between OCT angiography derived superficial vascular plexus (SVP) density for inter-eye differences, as well as for ganglion cell-inner plexiform (GCIPL) thickness, and visual outcomes in MS ON patients, providing insights into the interactions between retinal tissue and vascular changes. For immunological markers, Schmetzer et al. controversially question the pathogenic nature of the aquaporin 4 IgG antibody (AQP4-IgG) in NMOSD with the finding that Ab-positive patients showed no correlation between titer levels and clinical disease activity. Finally, Kang et al. report elevated serum levels of T-helper cell-related cytokines in patients with positive myelin-oligodendrocyte glycoprotein antibodies (MG-IgG), suggesting that Th17 cells may play a role in its autoimmunity.

The third theme is more directly concerned with clinical outcomes. Park et al. report the potential clinical utility of low-contrast visual acuity (2.5%) in detecting patients with prior ON, being superior to more conventional high-contrast visual acuity measures. Zhao et al. performed an observational study of low-dose rituximab treatment in NMO-ON, demonstrating good tolerance, reductions in aquaporin-4 antibody titers (*p* = 0.01), and good reductions in CD19 + B cells. For acute ON relapses in AQP4-IgG NMOSD, Akaishi et al. provide further evidence of the benefit of early systemic corticosteroid treatment in preserving visual acuity. Finally, Klumbies et al. reveal secondary analyzes from the SUPREME study (Sunphenon in Progressive Forms of MS, NCT00799890), which evaluates the effect of epigallocatechin-gallate (EGCG), an anti-inflammatory and antioxidant ingredient in green tea, on OCT. Unfortunately, they found no differences between the treated and placebo groups after 2 years for the peripapillary retinal nerve fiber layer and ganglion cell/inner plexiform layer thicknesses. Although the study may have been underpowered, it is important to investigate potential neuroprotective agents in progressive MS where there is a paucity of effective therapies.

In conclusion, this research topic contains articles that communicate the latest knowledge and developments from a wide range of conditions related to neuroinflammation affecting both the eye and the CNS visual system. Topics include understanding the pathophysiological mechanisms, studying imaging and immunological markers of the diseases, clinical outcomes, and exploring potential treatments. The experiments described range from animal models and cell-based tests to human *in vivo* studies. We hope that this collection of research work on this topic will help readers and researchers interested in seeing the eye and visual system as a model for dealing with inflammation from different angles. The breath of the works presented here enhance understanding across multiple disciplines and hopefully inspire innovative research that accelerates understanding of disease mechanisms and develops effective therapies.

## Author Contributions

GR wrote the draft of the editorial. CG and AT revised and corrected it. All authors contributed to the article and approved the submitted version.

## Conflict of Interest

The authors declare that the research was conducted in the absence of any commercial or financial relationships that could be construed as a potential conflict of interest.

## Publisher's Note

All claims expressed in this article are solely those of the authors and do not necessarily represent those of their affiliated organizations, or those of the publisher, the editors and the reviewers. Any product that may be evaluated in this article, or claim that may be made by its manufacturer, is not guaranteed or endorsed by the publisher.
